# Different origins and processing methods affect the intrinsic quality of ginger: a novel approach to evaluating ginger quality

**DOI:** 10.3389/fchem.2023.1296712

**Published:** 2023-11-10

**Authors:** Jie Wang, Yujie Liu, Chijing Zuo, Jing Zhang, Wanhui Liang, Yan Liu, Weidong Yu, Hao Yu, Can Peng

**Affiliations:** ^1^ A School of Pharmacy, Anhui University of Chinese Medicine, Hefei, China; ^2^ College of Traditional Chinese Medicine, Bozhou University, Bozhou, China; ^3^ MOE-Anhui Joint Collaborative Innovation Center for Quality Improvement of Anhui Genuine Chinese Medicinal Materials, Hefei, China; ^4^ Anhui Province Rural Revitalization Collaborative Technology Service Center, Hefei, China; ^5^ Anhui Province Modern Chinese Medicine Industry Common Technology Research Center, Heifei, China; ^6^ Anhui Province Key Laboratory of Pharmaceutical Preparation Technology and Application, Heifei, Anhui, China

**Keywords:** HPLC, high-performance liquid chromatography, SA, similarity analysis, PCA, principal component analysis, HCA, hierarchical cluster analysis, DA, discriminant analysis, SPSS, statistical product and service solutions, RPA, relative peak area, RRT, relative retention time

## Abstract

**Introduction:** Ginger *(Zingiber officinale Roce.)* is a widely consumed food item and a prominent traditional Chinese medicinal herb. The intrinsic quality of ginger may differ due to variations in its origin and processing techniques. To evaluate the quality of ginger, a straightforward and efficient discriminatory approach has been devised, utilizing 6-gingerol, 8-gingerol, and 10-gingerol as benchmarks.

**Methods:** In order to categorize ginger samples according to their cultivated origins with different longitude and latitude (Shandong, Anhui, and Yunnan provinces in China) and processing methods (liquid nitrogen pulverization, ultra-micro grinding, and mortar grinding), similarity analysis (SA), hierarchical cluster analysis (HCA), and principal component analysis (PCA) were employed. Furthermore, there was a quantitative determination of the significant marker compounds gingerols, which has considerable impact on maintaining quality control and distinguishing ginger products accurately. Moreover, discrimination analysis (DA) was utilized to further distinguish and classify samples with unknown membership degrees based on the eigenvalues, with the aim of achieving optimal discrimination between groups.

**Results:** The findings obtained from the high-performance liquid chromatography (HPLC) data revealed that the levels of various gingerols present in all samples exhibited significant variations. The study confirmed that the quality of ginger was primarily influenced by its origin and processing method, with the former being the dominant factor. Notably, the sample obtained from Anhui province and subjected to liquid nitrogen pulverization demonstrated the highest content of gingerols.

**Conclusion:** The results obtained from the analysis of SA, HCA, PCA, and DA were consistent and could be employed to evaluate the quality of ginger. As such, the combination of HPLC fingerprints and chemo metric techniques provided a dependable approach for comprehensively assessing the quality and processing of ginger.

## Highlights

Discriminant analysis of ginger source by 6-gingerol, 8-gingerol, 10 gingerol.
Different processing methods can be employed to address the problem of ginger’s loss of active ingredients during utilization or storage.
The fingerprint spectrum of ginger derived from different production areas was established through the use of HPLC and similarity software.

## 1 Introduction

Ginger (*Zingiber officinale* Roce.) is the rhizome of the ginger family and belongs to the perennial herb (Shukla and Singh, 2007) Ginger originates in Asia. Presently, this plant is commonly cultivated in tropical regions around the world as a spice and dietary supplement (Peng et al., 2017). Additionally, ginger is highly sought after in the perfume industry due to its abundance of volatile essential oils (Marak et al., 2019). As a result of the copious amounts of vitamins contained within ginger, it is commonly utilized as a flavoring component in candies and beverages (Sangwan et al., 2014). As per the research findings, the pungent taste of ginger is a result of the presence of shogaols and gingerols. Moreover, the unique flavor of ginger can be attributed to the volatility and pungency of its essential oil (Yamaguchi et al., 2010). Simultaneously, ginger is also a well-known traditional Chinese medicine (TCM) that has been widely utilized in clinical settings due to its medicinal and nutritional properties. As a medicine-food homology, ginger possesses various health benefits such as bacteriostasis, detoxification, anti-inflammatory properties, hypolipidemia, anti-oxidant properties, and tumor suppression (Shanmugam et al., 2011; Funk et al., 2016; Kim et al., 2018; Mahomoodally et al., 2021). The advantageous health properties of ginger have been attributed to an array of functional components, including gingerol, shogaol, phenolic elements, purine compounds, ginger oil, volatile oils, active polysaccharides, and glycoproteins (Levita et al., 2018). The primary constituent of ginger is gingerol, which comprises 6-gingerol, 8-gingerol, and 10-gingerol, with 6-gingerol constituting over 75% of the total composition (Liu et al., 2019). Gingerols convert into their corresponding shogaols after being subjected to long-term storage or heat treatment, resulting in the manifestation of high levels of antioxidant activity (Ballester et al., 2022). Nevertheless, the constituents and composition of ginger were substantially influenced by elements such as localities, climates, and soil environments (Gaur et al., 2016). These factors directly affect the unique flavor of ginger and its medical and health effects. The specific conditions of cultivation and various processing methods are crucial factors in determining the active constituents that define its distinct characteristics (Chen et al., 2019). In light of the consistent increase in the utilization and consumption of ginger, it is of utmost importance to establish an appropriate methodology for the development of quality control measures. The Chinese Pharmacopoeia (China Pharmacopoeia Committee, 2020) is identified 6-gingerol as the marker compound for evaluating the quality of ginger. However, ginger is composed of a multitude of intricate constituents beyond this singular compound. Consequently, relying solely on one component as the standard for quality evaluation is inadequate. It is therefore imperative to include more effective ingredients in the evaluation process, rather than relying on a single compound.

The utilization of chemometric methodologies, particularly principal component analysis (PCA) and hierarchical cluster analysis (HCA), has facilitated the systematic acquisition of chemical profiles that have a broad range of applications in the classification and identification of medicinal compounds. These techniques have been extensively employed in the analysis of the chemical composition of herbal medicines (Guo et al., 2018). Furthermore, the application of high-performance liquid chromatography (HPLC) in combination with multivariate statistical methods has been utilized to discriminate and classify various constituents (Miao et al., 2019). Chromatographic fingerprinting was considered as a reliable method to evaluate the quality of traditional Chinese medicine (Cui et al., 2016). In addition, the TCM fingerprint is internationally recognized for the evaluation and quality control of TCM and related products.

To summarize, the constituents of gingerols involved in processing have the potential to greatly affect the ultimate innate excellence of ginger. The established methods for evaluating quality do not suffice in meeting the needs of industry progress and quality appraisal. The present study employs a variety of chemometric methods, such as SA, HCA, PCA, and DA, to analyze the HPLC fingerprint profiles of ginger, with a particular emphasis on the active substances of 6, 8, and 10-gingerols, in order to evaluate the quality of ginger. Furthermore, standard samples were utilized to determine the contents of markers. Through the integration of stoichiometric techniques and fingerprint analysis, the influence of the production area and processing processes on the intrinsic quality of ginger was systematically examined.

## 2 Materials and methods

### 2.1 Chemicals

**FIGURE 1:**
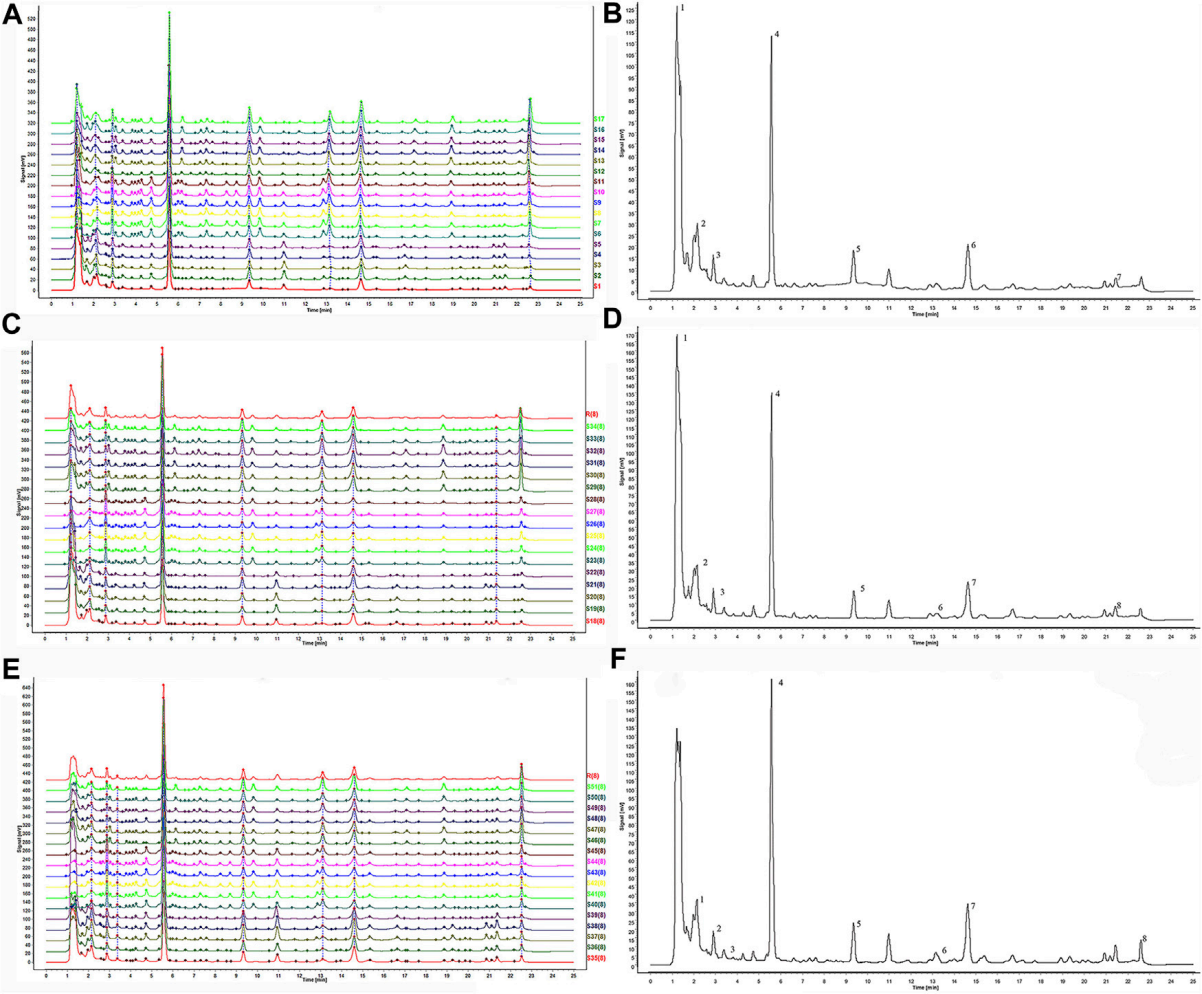
The HPLC fingerprints of *Zingiber officinale* Roce. and the characteristic spectrogram for the chromatographic fingerprint of *Zingiber officinale* Roce. **(A)** The fingerprints of superfine crush pre-treated ginger. **(B)** The reference standard fingerprint of superfine crush pre-treated ginger. **(C)** The fingerprints of grinding pretreatment ginger. **(D)** The reference standard fingerprints of grinding pretreatment ginger. **(E)** The fingerprints of liquid nitrogen freeze mill pre-treated ginger. **(F)** The reference standard fingerprints of liquid nitrogen freeze mill pre-treated ginger.

Ginger reference substance: 6-gingerol (lot number: 111833–201806, purity >99.9%) was purchased from National Institutes for Food and Drug Control, 8-gingerol (lot number: CHB 180305, purity >98%) and 10-gingerol (lot number: CHB 180311, purity >98%) were purchased from Chengdu Chroma-Biotech Co., Ltd. Their structures were shown in Figure 1. The deionized water was generated by a laboratory water purification system (HITECH Instruments CO., Ltd). The acetic acid was of analytical grade purchased from Shanghai Runjie Chemical Reagent Co. Ltd. Other chemicals, like methanol (lot number: 34860-4L-R) and acetonitrile (lot number: 134752), were HPLC grade and obtained by Sigma-Aldrich Shanghai Co, Ltd. and Beijing J&K Scientific Ltd., respectively.

### 2.2 Ginger samples

Three kinds of ginger samples were collected from the south (Jietou town, Qujing city, Yunnan province), middle (Tianmen Town, Tongling City, Anhui Province) and north (Tancheng County, Linyi City, Shandong Province) of China, respectively (Table 1). In our work, 5 batches of ginger from Shandong, 6 batches of ginger from Anhui and 6 batches of ginger from Yunnan were used in the experiment. Professor Xie Dongmei has authenticated all samples from the Department of Pharmacy of Anhui University of Chinese medicine (Anhui, China). They were identified as the dried rhizome of ginger and complied with official Chinese pharmacopeia (China Pharmacopoeia Committee, 2020).

**TABLE 1:** A summary of the tested sample.

Sample no.	Sources	Pretreatment	Longitude and latitude
S1-S5	Tancheng County, Linyi City, Shandong Province	Superfine	Longitude: 118.35; Latitude: 34.62
S6-S11	Tianmen Town, Tongling City, Anhui Province	Superfine	Longitude: 117.84; Latitude: 30.83
S12-S17	Jietou Town, Qujing City, Yunnan Province	Superfine	Longitude: 98.13; Latitude: 25.17
S18-S22	Tancheng County, Linyi City, Shandong Province	Grinding	Longitude: 118.35; Latitude: 34.62
S23-S28	Tianmen Town, Tongling City, Anhui Province	Grinding	Longitude: 117.84; Latitude: 30.83
S29-S34	Jietou Town, Qujing City, Yunnan Province	Grinding	Longitude: 98.13; Latitude: 25.17
S35-S39	Tancheng County, Linyi City, Shandong Province	Liquid nitrogen	Longitude: 118.35; Latitude: 34.62
S40-S45	Tianmen Town, Tongling City, Anhui Province	Liquid nitrogen	Longitude: 117.84; Latitude: 30.83
S46-S51	Jietou Town, Qujing City, Yunnan Province	Liquid nitrogen	Longitude: 98.13; Latitude: 25.17

### 2.3 Sample preparation

First of all, scissors were used to cut haphazardly selected ginger from every origin into thin strips. Then strips were pulverized to powder samples through the following three methods (Table 1). 1) Strips were chilled into liquid nitrogen, then pulverized with a magnetically driven impactor to let it turn into a fine powder. This experiment was performed by the SPEX SamplePrep’s Freezer/Mills. The instrument condition was as follows: run time, 1 min; cycles, 2; rate, 8 cps. 2) Microchips were put into the ST-528 Ultra-micro Crusher (Ruian saite electromechanical Co., Ltd.) and removed the fine powder that flies into the upper container. 3) Ginger was placed into the mortar and ground it manually in one direction. According to the requirements of Chinese Pharmacopoeia 2015, powders passed through 50 mesh sieves were selected. All of the steps were performed at 25°C.

The scale was used in one out of 10,000 g of precision. The ginger powder was precisely weighted about 0.25 g and placed in a core-shaped flask. Moreover, the powder was added with 75% chromatogram grade methanol (20 mL). The total weight of the weighing samples and container were weighed and written down. After 40 min of ultrasonic treatment, with 30°C water in the power of 300 W and the frequency was 40 kHz. After ultrasonic, the ginger extracting solution was naturally cooled. The whole weight was weighed again to make up the lost weight with 75% chromatogram grade methanol. Finally, the solution was filtrated through a 0.22 μm microporous filter membrane and the filtration was taken as the test solution. All solutions and powders were placed in a light-proof sealed plastic bag, numbered and stored in the refrigerator at −20°C.

### 2.4 Apparatus and chromatographic conditions and optimization

The Waters Acquity UPLC™ system was equipped with an auto-sample, a binary pump, a degasser, the 4-channel solvent degasser integrated into the pump module, UV-Vis variable wavelength detector, thermostated column compartment, and connected to Waters Empower 2 software. A reverse-phase column (C_18_ analytical column, 4.6 mm × 150 mm, 5 μm, WondaCract ODS-2, Japan) was carried out in the analysis. The best separation effect was obtained in the chromatogram.

By comparing different mobile phase compositions and different mobile phase proportions and gradient conditions, the optimal choice was finally determined. The mobile phase was composed of water containing 0.1% (v/v) acetic acid (A) and acetonitrile (B). The gradient program for the HPLC was as follows: 0–15 min, 51%–70% B; 15–20 min, 70%–90% B; 20–21 min, 90%–51% B; 21–24 min, 51% B. The column and the auto-sampler temperature were maintained at 25°C. The sample injection volume was 20 μL. The UV spectrum was performed and peak absorbance was detected at 280 nm.

### 2.5 Chemometrics analysis

#### 2.5.1 Similarity analysis

Chromatograms can make representative of all samples and have the characteristics of completeness and ambiguity. By analyzing the mutual mode of the chromatogram, the sample could be well-identified and analyzed. The contents of several chemical components were different. It is necessary to find possible quality markers to identify and analyze different samples (Tan et al., 2018). Ginger, in the same pretreatment, was categorized into a team. The HPLC chromatograms of the ginger were exported as CDF files. They imported them into a software named Similarity Evaluation System for Chromatographic Fingerprint of Traditional Chinese Medicine. The software was validated and supplied by the Chinese Pharmacopoeia Committee (Version 2012.130723; Beijing, China) and analyzed the data under optimal conditions. The software calculates the mean values and generates mock chromatograms which were used as representative standard chromatograms for the ginger samples. Correlation coefficients and similarities between the means were calculated for each mock chromatogram.

#### 2.5.2 Hierarchical cluster analysis

Hierarchical cluster analysis (HCA) was a multivariate analysis technique that categorized samples to show the degree of correlation between a large number of samples (L et al., 2011). Each object was the same as the other objects in a group but differed from the other objects regarding predetermined selection criteria (Kong et al., 2009). Based on the subjective comparison, HCA was used to evaluate the chromatographic peaks areas of active ingredients.

#### 2.5.3 Principal component analysis

Principal component analysis (PCA) can remove redundant information, reduce multidimensional data to lower dimensions, and highlight hidden features (Liu et al., 2016; Chen et al., 2017; Guo et al., 2018; Ghisoni et al., 2019). Here, SIMCA software was used to evaluate and select the similarity evaluations, get more accurate and detailed fingerprint information. By importing pre-processed fingerprints spectrum data to the SIMCA system, quality control and ginger evaluation from different origins were established.

#### 2.5.4 Discriminant analysis

To further trace the origin of ginger and realize the source identification of ginger. Discriminant analysis (DA) was a standard method to identify the sample type in multivariate statistics. Fisher discrimination was used in Statistical Product and Service Solutions (SPSS).

## 3 Results and discussion

### 3.1 Establishment of HPLC fingerprint

The proportion of the acetonitrile phase was decreased to make the peak time earlier. Under the conditions reported in the literature, acetic acid was added to the water phase. The degree of sample separation was tested at different concentrations of the acetonitrile and water phase. Finally, acetonitrile and 0.1% acetic acid were selected as the mobile phase. Gradient elution displayed a better resolution and made each component peak well separated from adjacent peaks.

The similarity evaluation system had excellent resolution and large areas in the HPLC fingerprints, which could be regarded as the reference chromatogram to identify ginger. According to the fingerprints analysis, seven common peaks were demarcated from superfine samples (Figure 1A). Eight peaks were determined from grinding and liquid nitrogen samples of 17 batches of ginger, respectively (Figures 1C, E). In the three treatments, the common peaks obtained all contain 6-gingerol, 8-gingerol, and 10-gingerol.

### 3.2 Similarity analysis

The system separately compared all the 17 batches of each team (Figures 1A, C, E) to generate a reference chromatogram. The median method was utilized to compare the similarities between the entire and reference chromatogram. Differences in correlation coefficients indicate the variation of the fingerprint and quality of these samples (Zhong et al., 2015; Li et al., 2016). It indicated that it is necessary to identify all the possible quality markers to distinguish and analyze different samples. In this study, the correlation coefficient close to 1.0 indicated that the samples were entirely similar by multivariate analyzing score and loading.

Table 1 showed correlation coefficients of 17 samples of ginger with different pretreatments, respectively. As shown in the similarity of grinding samples (Supplementary Table S1A), S6 and S7, S6 and S8, S6 and S9, S6, and S10 showed a high degree of similarity. S4 and S6, S4 and S7, S4 and S8, S4 and S9, S4, and S10 had low similarity (about 0.42). In Figure 1B, 7 related peaks may affect the quality of ginger. Peaks 2, 4, 5, 6, and 7 with the highest content as characteristic peaks were significant for quality control of ginger fingerprints. Meanwhile, similar characteristic peaks were obtained in the grinding samples. In Supplementary Table S1B, S23 and S24, S23, and S25, S23, and S27 correlated the grinding samples. There were eight peaks with the highest content in S23, which might affect the quality of samples. In contrast, the correlation between S20 and S23, S20 and S24, S20 and S25, and S20 and S27 were lower than other batches. Apparently, the contents of peak 4 in S20 were significantly higher than that of S23, 24, 25, and 27. Meanwhile, the content of peaks 5 and 7 of S20 was markedly higher than that of S23, S24, S25, and S27. As the control spectrum of grinding pretreatment samples shows (Figure 1D), peaks 4, 5, 7, and other characteristic peaks had crucial implications for quality control. Meanwhile, liquid nitrogen samples (Supplementary Table S1C) showed that S50 and S46, S50 and S48, S50, and S49, S50, and S51 had higher correlations. On the contrary, correlations between S35 and S42, S35, and S43, S35, and S44, S35, and S45 were lower than other batches. The content of peaks 2, 4, and 5 in S50 was lower than in other samples. The contents of peaks 2, 5, and 7 in S35 were quite different from those in S42, S43, and S45. It was observed that peaks 2, 4, 5, and 7 could significantly affect the quality of gingers (Figure 1F).

The total content of the main component had an essential effect on the intrinsic quality of ginger through the analysis of similarity data. Meanwhile, some small elements also showed significant differences in different samples. It was noted that other origin areas and processing methods influenced the quality of ginger samples.

### 3.3 Hierarchical cluster analysis

The dendrogram plot defined three distinctive clusters. These results showed that HCA could effectively distinguish samples from the same origin with different extraction methods and classify them into three quality clusters based on morphological characteristics.

**FIGURE 2:**
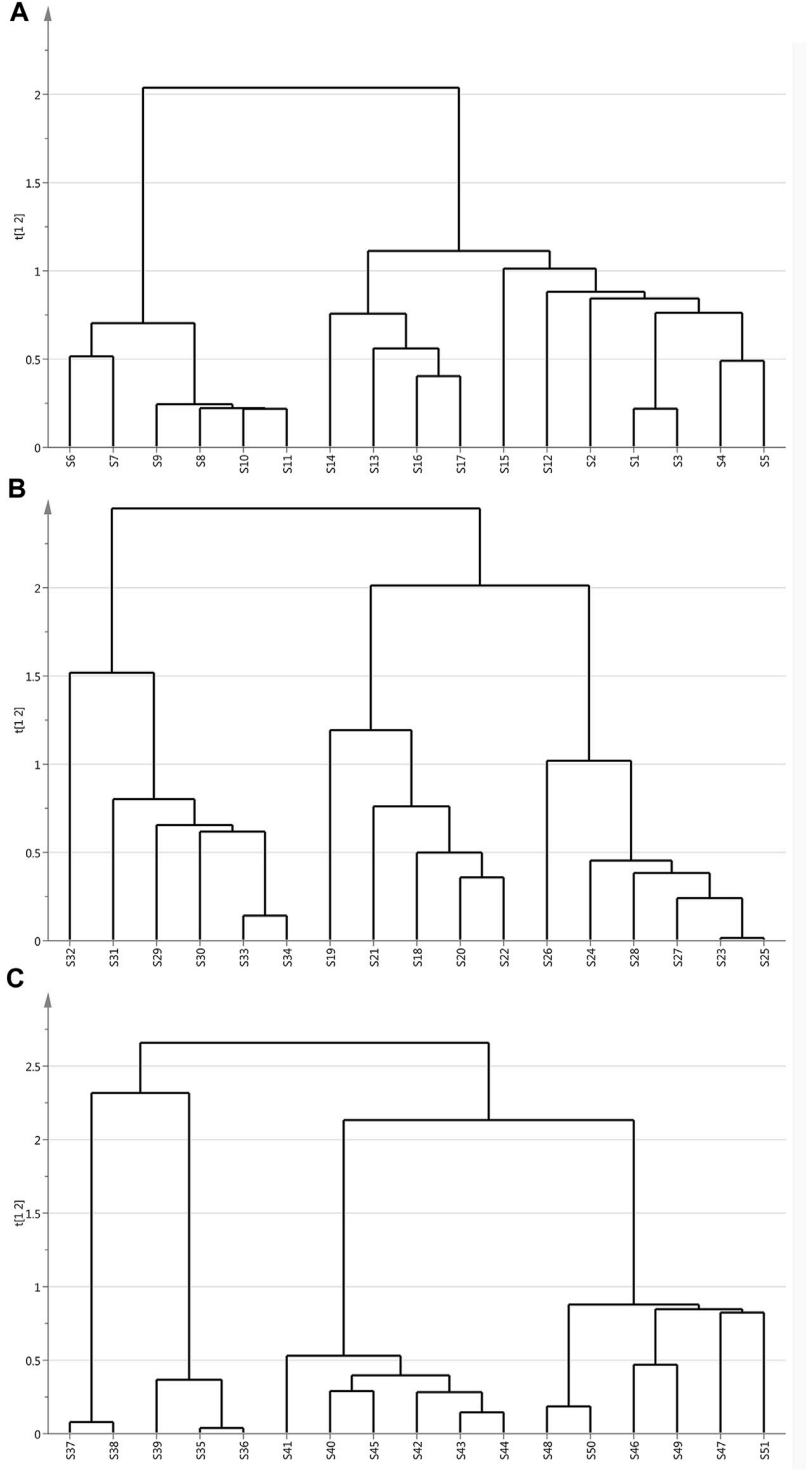
Dendrogram of ginger samples with different extraction methods: **(A)** Superfine crush pre-treated ginger. **(B)** Grinding pretreatment ginger. **(C)** Liquid nitrogen freeze mill pre-treated ginger.

**FIGURE 3:**
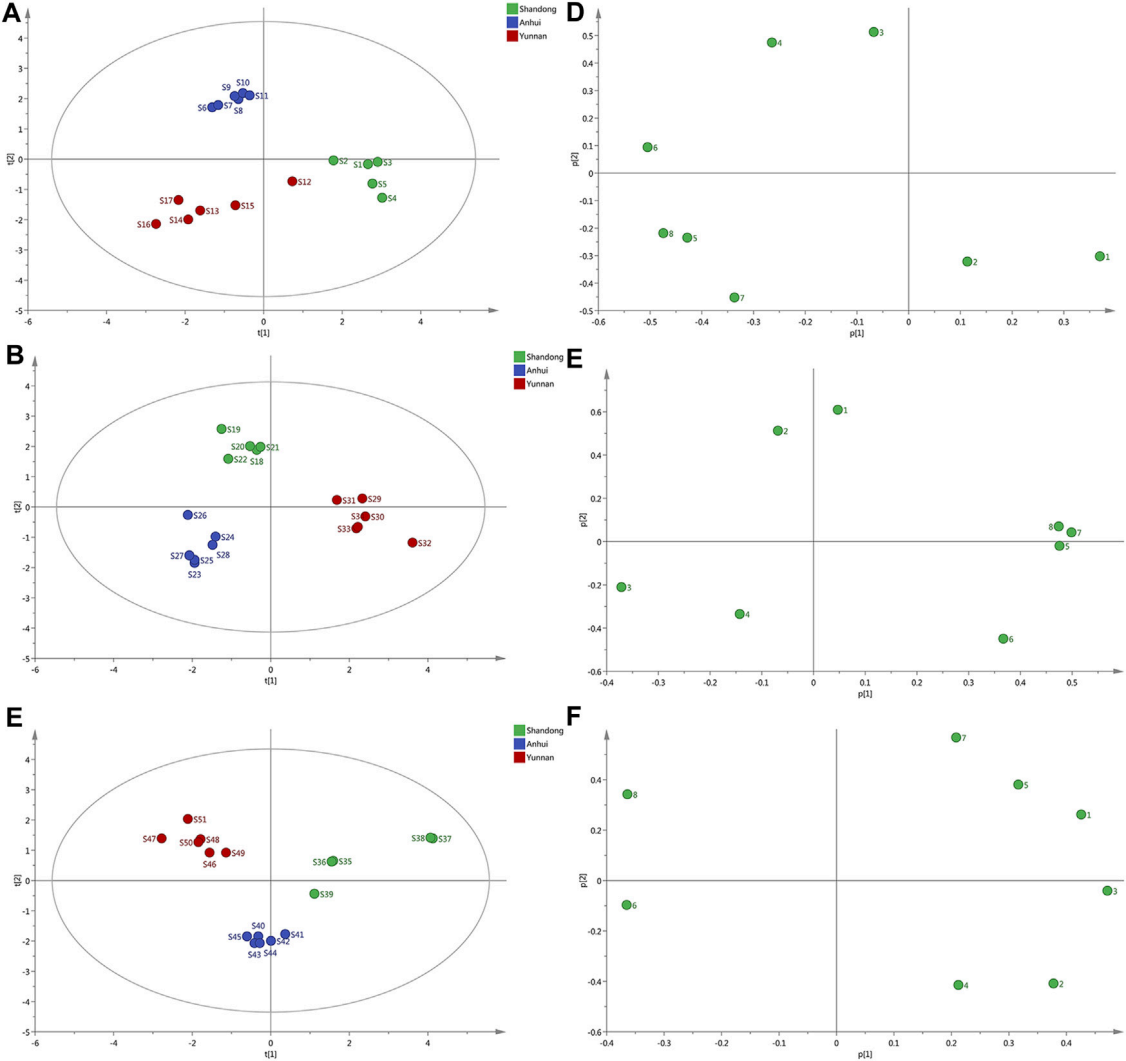
PCA score scatter plots and loading scatter plots for the samples. **(A–C)** The score scatter. Plots of ginger with different extraction methods from Shandong, Anhui, Yunnan, respectively. **(D–F)** The loadings plot of ginger with varying forms of extraction from Shandong, Anhui, Yunnan, respectively.

As shown in the scoring chart of ultramicro comminuted sample (Figure 2A), it could be classified into three separate groups. S6-S11 clustered into one category. The S13, S14, S16, S17 from Yunnan province were close to each other, they were regarded as one group. The profile of S12 and S15 were on the brink of S1-S5 samples. Therefore, S1-S5, S12, S15 were tended to be grouped into the same cluster. These inspired that the samples from Shandong and Yunnan province had similarities in chemical composition. Figures 2B, C showed the data of liquid nitrogen and grinding samples divided into three clusters based on different origins. According to the origins, 17 batches of samples were divided into three clusters strictly. This indicates that they differ in the content and distribution of the main chemical components. In Figure 3B, these were the compounds that separated S29-S34 as group 1. Group 2 was comprised of peak 18–22 and the samples were all collected from Shandong province. When it comes to the other six samples, S23-S28 from Anhui province was clustered in group 3. As shown in liquid nitrogen freezer mill pre-treated samples (Figure 2C), group 1 consisted of S35-S39 collected from Shandong province. Group 2 included samples S40-S45 collected from Anhui province. The extra six samples, S46-S51 from Anhui province, were clustered in group 3.

### 3.4 Principal component analysis

In Figure 3A, the samples could be visually classified into three separate groups according to different origins. S1-S5 were prone to be classified into one group. S6-S11 seemed to be grouped into the same cluster. What is more, S12-S17 were in the same group. The profile of S12 was closed to that of S1-S5 samples. To compare the result which obtained from both techniques, the PCA and HCA showed comparable results. Most samples with the same origin were aggregated into the same clusters. As shown in the PCA score plot (Figure 3B), the samples could be visually classified into three separate groups. S18-S22 (Shandong province) were in the same domain. S23-S28 (Anhui province) was also in the same domain. In comparison, the other six samples (S29-S34 from Yunnan province) were in the same domain. The dendrogram (Figure 3C) visibly showed that all samples were divided into three clusters. Five samples (S35-S39) from Anhui were classified into the same group. Indeed, samples from Anhui province (S40-S45) were clustered into the same group. The rest of the samples (S46-S51) formed a group. The results were consistent with their native place. It shows that the ginger from Anhui and the other two origins (Shandong and Yunnan province) were divided into three main categories.

The results of the principal component analysis were presented in the form of a score. The load focuses on multivariate changes that affect the differences between samples. Therefore, common peak areas of various resolved components were analyzed, and a data matrix of elements for samples was established (Kong et al., 2009). The peak areas of 8 components in each sample were the first and second PCs (PC1, PC2) for visually this new data matrix.

The loadings plot of ginger with different extraction was illustrated (Figure 3D–F). Analysis of the loading plot of PC1 against PC2 (Figure 3D) revealed that peaks contributing to PCA influenced the cluster in top-down order. Peaks 3, 4, and 6 that mainly contributed to PC2 classified S6-S11 as the same group. In summary, since PC1 contributing fewer peaks than PC2, samples on the left side of Figure 3D were presumed higher quality. Accordingly, ginger collected from Anhui province had the highest quality. The loadings scores indicated that the peaks 3, 4, 5, 6, 7 might be the main influencing factors for the discrimination of different samples. Analysis of ginger loading plots of PC1 against PC2 (Figure 3E) revealed that peaks explained by PC1, peaks 5, 7, and 8 influenced the cluster in top-down order. Owing to the higher intensities of peaks 7, 8, 5, Yunnan samples were characterized primarily by positive values of PC1. From the loading plot, peak 1, 2, 4, 5, 7, 8 had the most substantial influence on the quality of geographical differences and various processing techniques. From the loading plot of liquid nitrogen (Figure 3F), peaks 1, 3, 5, and 7 had an enormous impact on geographic difference quality. Besides, peak 2 and peak 4 also affect quality evaluation.

By determining the contents of those chemical marker compounds, the quality of gingers from various sources could be measured. The consistent result was obtained from SA, PCA, and HCA, implying that PCA was an efficient complement for HCA and SA. In consideration of our existing standard and the currently contained standards produced in the laboratory, 6-gingerol, 8-gingerol, 10-gingerol (peak 4, 5, 6 in superfine samples; peak 4, 5, 7 in grinding and liquid nitrogen samples) were selected as the natural substance to study the effect of producing area on the quality of ginger. Meanwhile, gingerols exhibited major medicinal activity as the main component of ginger.

### 3.5 Method validations of quantitative analysis

#### 3.5.1 Linearity

**FIGURE 4:**
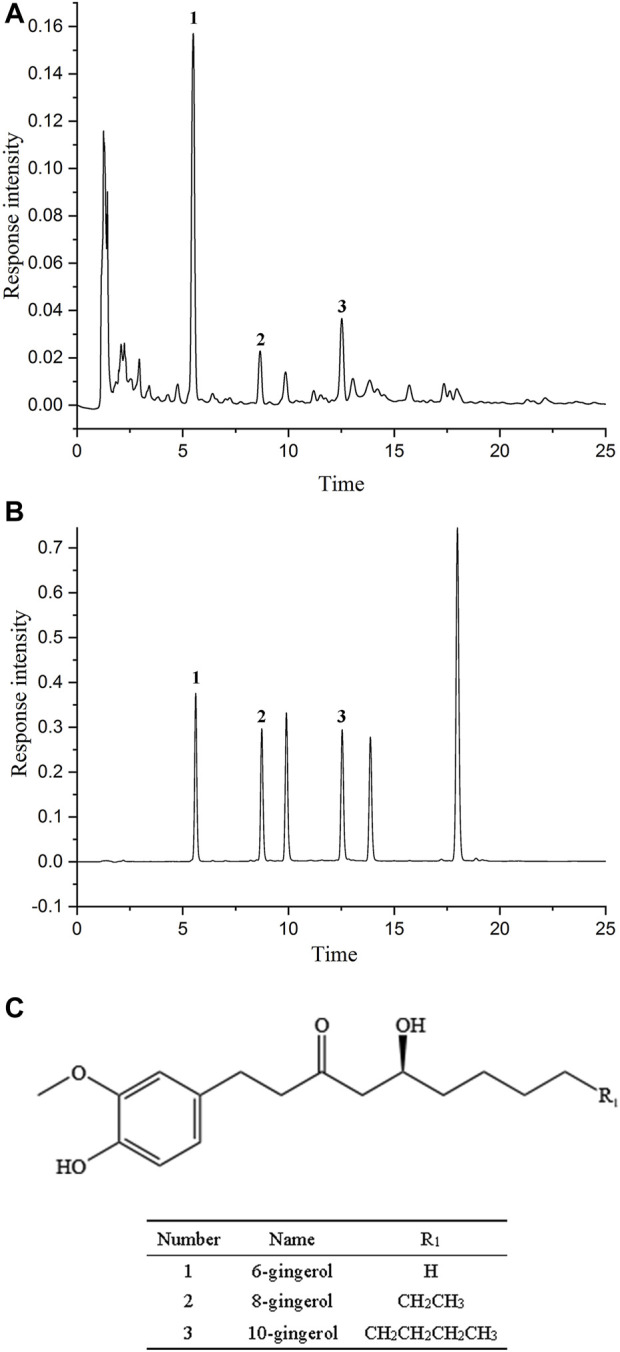
The chromatographic fingerprint and chemical structure of gingerols. **(A)** The chromatographic fingerprint of *Zingiber officinale* Roce. **(B)** The HPLC chromatogram of gingerols composition. (1) 6-gingerol; (2) 8-gingerol; (3) 10-gingerol. **(C)** The chemical structure of gingerols.

As shown in Figures 4A, B, methanol was used to prepare the mixed standard of 3 main ingredients at each concentration level to establish the calibration curves, ensuring that the concentration of samples was within the linear range. Table 2 demonstrated that the established calibration curves had a favorable direct relationship within the test ranges. The correlation coefficients were more excellent than 0.9990. Their structures are shown in Figure 4C.

**TABLE 2:** Calibration curve of three primary active ingredients in ginger samples.

	Calibration curve	Linear range (μg/mL)	Correction coefficient (R)
6-gingerol	Y = 18355x-25608	37.5–100	0.9996
8-gingerol	Y = 12277x-3811	4–30	0.9990
10-gingerol	Y = 10566x-876	30–60	0.9990

7Y, peak area; x, the concentration of each reference compound (μg/mL); R, the correlation coefficient of regression equations.

#### 3.5.2 Precision, repeatability, and stability

The standard sample precision was inspected six times of injection in succession. Six consecutive doses of the sample were performed for accurate inspection. Reproducibility was evaluated by comparing the peak areas of three independently prepared samples. The reproducibility assessed by comparing the peak area of three individually designed samples can be viewed in Supplementary Table S2. The methodology validation that the relative standard deviation (RSD) for precision, repeatability. The stability coefficient was lower than 3.37. All these results showed that the HPLC method is accurate and valid.

### 3.6 Contents analyses of the three primary active ingredients

The relative peak area (RPA) and relative retention time (RRT) was represented as the reference peaks. The contents of three primary active ingredients of ginger in Supplementary Table S3. It illustrated that the content of gingerols was significantly varied in each sample, especially the samples from different sources. The internal quality relationship should be evaluated to conquer the suitable origin and extraction method. The content of the 6-gingerol was the most abundant component. The sequence of the total contents of the three compounds in diverse samples was 6-gingerol >8-gingerol >10-gingerol. They were primary index ingredients as well as potentially active compounds in ginger. Consequently, the quality control approach in Chinese Pharmacopoeia using only 6-gingerol for quantification was improper. It was suggested that other gingerols (8-gingerol and 10-gingerol) also should be quantified. Hence, we used 6-gingerol as well as 8-gingerol and 10-gingerol as the quality marker in this paper.

The samples collected from Anhui province had the highest total contents of three components. It indicated that the ginger from the Anhui province might have the most excellent exceptional quality. Moreover, the samples crushed by low-temperature liquid nitrogen, the content of gingerols, were significantly higher than other pre-processing crushing methods. Liquid nitrogen processing might provide the usable method to get the highest gingerols contents of ginger.

### 3.7 Discriminant analysis

Based on Fisher’s linear discriminant function coefficients, classification discriminant models of samples were established from three origins: Shandong, Anhui, and Yunnan provinces (*X*_*1:*_ 6-gingerol, *X*_*2:*_ 8-gingerol, *X*_*3:*_ 10-gingerol,):

In superfine samples:

$$\text{Shandong}=0.193X_{1}-0.256X_{2}+0.639X_{3}-59.935$$ (1)

$$\text{Anhui}=0.184X_{1}-0.176X_{2}+0.770X_{3}-78.441$$ (2)

$$\text{Yunnan}=0.169X_{1}-0.087X_{2}+0.595X_{3}-57.403$$ (3)

In grinding samples:

$$\text{Shandong}=-0.116X_{1}+0.087X_{2}+1.481X_{3}-62.072$$ (4)

$$\text{Anhui}=-0.700X_{1}+1.815X_{2}+2.829X_{3}-207.383$$ (5)

$$\text{Yunnan}=0.360X_{1}-1.173X_{2}+0.305X_{3}-32.612$$ (6)

In liquid nitrogen samples:

$$\text{Shandong}=0.076X_{1}+1.484X_{2}+0.127X_{3}-69.835$$ (7)

$$\text{Anhui}=0.028X_{1}+1.539X_{2}+0.196X_{3}-68.114$$ (8)

$$\text{Yunnan}=0.187X_{1}+1.125X_{2}-0.053X_{3}-71.096$$ (9)

When discriminating against the samples type, the samples were plugged into the three classification functions, respectively. Then classify them into the class corresponding to the most considerable numerical value as the substantial value. X represents the variable. Meanwhile, the regression estimate method was used for discriminant analysis. The actual result was shown in Table 3, 82.4%, 100%, and 88.2% of the original grouped cases were correctly classified. It showed that this method was practical and feasible. The discriminant analysis could achieve the prediction and identification of the origin of gingers. So as to, discriminant analysis had an absolute value for the promotion and application.

**TABLE 3:** The percent of correctly classified original grouped.

Groups	Correctly classified (%)
superfine samples	82.4
grinding samples	100.0
liquid nitrogen samples	88.2

## 4 Discussion

Interestingly, a discriminant analysis of the region of origin of ginger found that the retention of active ingredients varied among different processing methods. In order to investigate potential factors, the ginger was ground using liquid nitrogen freezing, resulting in the smallest particle size. Additionally, the use of lower temperatures helped to preserve the stability of the phenolic components. Simultaneously, the elevated temperature involved in the ultra-fine grinding procedure, coupled with the excessive powder produced during the grinding and crushing stages, has the potential to diminish the concentration of active constituents. The gingerols may convert into shogaols upon exposure to high temperatures during the drying or frying process. Consequently, the anti-oxidant and antimicrobial properties of gingerols are enhanced. In addition, it has been established that practical processing methods have a substantial influence on the gingerol content. The results of the current study suggest that the utilization of liquid nitrogen freezing is a superior process for achieving the highest recovery of gingerol from ginger (Chen et al., 2019). The application of ultrasonic pressure waves and cavitation effectively disrupted the cells and cell walls, leading to the breakdown of the ginger material. Furthermore, the utilization of ultrasound pretreatment has the added advantage of preserving the natural color of dried ginger slices. These techniques have been shown to be effective in enhancing the concentration of bioactive compounds such as gingerols oil, derivatives, and antioxidants, while also inactivating harmful enzymes like polyphenol oxidase and peroxidase (Osae et al., 2019). The temperature was an essential factor to be considered for determining the optimal extraction condition (Hsieh et al., 2020). The source of ginger is identified, and the advantages and disadvantages of ginger quality are evaluated through this method. The liquid nitrogen freezing and crushing method and ultrasonic extraction method retain the unique flavor of ginger to make a relevant diet. The evaluation method for ginger will serve as a potential and valuable reference for evaluating the quality, extraction methods, and development of food or other related traditional Chinese medicines. It is necessary to acknowledge the limitations of this work, for example, the number of ginger samples is small and all compounds in ginger were not acknowledged through the HPLC. In our future work, we intend to collect more ginger samples and identify more compounds of ginger by mass spectrometry.

## 5 Conclusion

In this work, a fast and straightforward HPLC fingerprint of ginger was developed. The results of chemometric techniques such as SA, HCA, PCA, and DA were suitable for analyzing fingerprint data, establishing quality control, and evaluating ginger. Characteristic fingerprint peaks were identified, and gingerol components could be used as markers and quantitatively determined. Based on the analysis, the disparity of samples from different origins was the dominant factor in quality differences and geographical distance. The physicochemical properties of ginger are greatly influenced by the processing, fermentation, and drying methods employed. In essence, Anhui serves as the most suitable source of ginger, while the optimal processing technique involves the use of liquid nitrogen to freeze and crush the ginger. These findings provide valuable insights for the future utilization of ginger. For scholars conducting research on food and drug quality control of traditional Chinese medicine, this series of development strategies can offer constructive suggestions and ideas.

## Data Availability

The original contributions presented in the study are included in the article/Supplementary Material, further inquiries can be directed to the corresponding authors.

## Abbreviations

HPLC, high-performance liquid chromatography; SA, similarity analysis; PCA, principal component analysis; HCA, hierarchical cluster analysis; DA, discriminant analysis; SPSS, statistical product and service solutions; RPA, relative peak area; RRT, relative retention time.
