# Supplementary material for: Different origins and processing methods affect the intrinsic quality of ginger: a novel approach to evaluating ginger quality
Source: Front Chem. 2023 Nov 10;11:1296712. doi: 10.3389/fchem.2023.1296712 (PMC10667423; doi:10.3389/fchem.2023.1296712)
Supplement: Supplementary file 1 [file Table1.DOCX]

**Table S1.** Similarities of the chromatograms of *Zingiber officinale* Roce.samples based on the correlation coefficients. (A)superfine crush pre-treated ginger. (B)grinding pretreatment ginger. (C)Liquid nitrogen freeze mill pre-treated ginger. (D)samples from Anhui province.

A

|  | S1 | S2 | S3 | S4 | S5 | S6 | S7 | S8 | S9 | S10 | S11 | S12 | S13 | S14 | S15 | S16 | S17 | S |
| --- | --- | --- | --- | --- | --- | --- | --- | --- | --- | --- | --- | --- | --- | --- | --- | --- | --- | --- |
| S1 | 1 |  |  |  |  |  |  |  |  |  |  |  |  |  |  |  |  |  |
| S2 | 0.995 | 1 |  |  |  |  |  |  |  |  |  |  |  |  |  |  |  |  |
| S3 | 0.983 | 0.987 | 1 |  |  |  |  |  |  |  |  |  |  |  |  |  |  |  |
| S4 | 0.988 | 0.983 | 0.982 | 1 |  |  |  |  |  |  |  |  |  |  |  |  |  |  |
| S5 | 0.995 | 0.993 | 0.984 | 0.997 | 1 |  |  |  |  |  |  |  |  |  |  |  |  |  |
| S6 | 0.470 | 0.477 | 0.547 | 0.425 | 0.430 | 1 |  |  |  |  |  |  |  |  |  |  |  |  |
| S7 | 0.461 | 0.467 | 0.539 | 0.416 | 0.421 | 1 | 1 |  |  |  |  |  |  |  |  |  |  |  |
| S8 | 0.457 | 0.464 | 0.54 | 0.413 | 0.418 | 0.995 | 0.996 | 1 |  |  |  |  |  |  |  |  |  |  |
| S9 | 0.469 | 0.485 | 0.552 | 0.423 | 0.431 | 0.989 | 0.990 | 0.990 | 1 |  |  |  |  |  |  |  |  |  |
| S10 | 0.473 | 0.481 | 0.553 | 0.431 | 0.436 | 0.994 | 0.994 | 0.995 | 0.990 | 1 |  |  |  |  |  |  |  |  |
| S11 | 0.740 | 0.756 | 0.805 | 0.709 | 0.714 | 0.918 | 0.915 | 0.916 | 0.925 | 0.921 | 1 |  |  |  |  |  |  |  |
| S12 | 0.785 | 0.788 | 0.845 | 0.765 | 0.768 | 0.829 | 0.826 | 0.829 | 0.825 | 0.830 | 0.929 | 1 |  |  |  |  |  |  |
| S13 | 0.826 | 0.824 | 0.864 | 0.812 | 0.811 | 0.806 | 0.803 | 0.797 | 0.801 | 0.808 | 0.921 | 0.973 | 1 |  |  |  |  |  |
| S14 | 0.806 | 0.803 | 0.844 | 0.790 | 0.790 | 0.809 | 0.806 | 0.800 | 0.807 | 0.804 | 0.913 | 0.967 | 0.991 | 1 |  |  |  |  |
| S15 | 0.876 | 0.875 | 0.900 | 0.862 | 0.864 | 0.743 | 0.738 | 0.730 | 0.745 | 0.739 | 0.900 | 0.942 | 0.984 | 0.986 | 1 |  |  |  |
| S16 | 0.883 | 0.881 | 0.901 | 0.860 | 0.866 | 0.752 | 0.746 | 0.733 | 0.747 | 0.743 | 0.898 | 0.942 | 0.983 | 0.978 | 0.991 | 1 |  |  |
| S17 | 0.782 | 0.781 | 0.833 | 0.760 | 0.759 | 0.886 | 0.884 | 0.876 | 0.877 | 0.881 | 0.958 | 0.966 | 0.979 | 0.975 | 0.957 | 0.962 | 1 | 0.976 |
| S | 0.886 | 0.889 | 0.923 | 0.864 | 0.868 | 0.814 | 0.809 | 0.806 | 0.813 | 0.815 | 0.956 | 0.958 | 0.974 | 0.964 | 0.973 | 0.976 | 0.976 | 1 |

B

|  | S18 | S19 | S20 | S21 | S22 | S23 | S24 | S25 | S26 | S27 | S28 | S29 | S30 | S31 | S32 | S33 | S34 | S |
| --- | --- | --- | --- | --- | --- | --- | --- | --- | --- | --- | --- | --- | --- | --- | --- | --- | --- | --- |
| S18 | 1 |  |  |  |  |  |  |  |  |  |  |  |  |  |  |  |  |  |
| S19 | 0.990 | 1 |  |  |  |  |  |  |  |  |  |  |  |  |  |  |  |  |
| S20 | 0.999 | 0.986 | 1 |  |  |  |  |  |  |  |  |  |  |  |  |  |  |  |
| S21 | 0.992 | 0.994 | 0.989 | 1 |  |  |  |  |  |  |  |  |  |  |  |  |  |  |
| S22 | 0.994 | 0.996 | 0.990 | 0.999 | 1 |  |  |  |  |  |  |  |  |  |  |  |  |  |
| S23 | 0.451 | 0.493 | 0.436 | 0.555 | 0.533 | 1 |  |  |  |  |  |  |  |  |  |  |  |  |
| S24 | 0.494 | 0.541 | 0.479 | 0.596 | 0.576 | 0.997 | 1 |  |  |  |  |  |  |  |  |  |  |  |
| S25 | 0.492 | 0.539 | 0.477 | 0.595 | 0.575 | 0.998 | 1 | 1 |  |  |  |  |  |  |  |  |  |  |
| S26 | 0.498 | 0.564 | 0.480 | 0.604 | 0.586 | 0.976 | 0.987 | 0.987 | 1 |  |  |  |  |  |  |  |  |  |
| S27 | 0.481 | 0.527 | 0.466 | 0.584 | 0.563 | 0.999 | 0.999 | 1 | 0.985 | 1 |  |  |  |  |  |  |  |  |
| S28 | 0.534 | 0.569 | 0.520 | 0.63 | 0.609 | 0.995 | 0.995 | 0.995 | 0.971 | 0.995 | 1 |  |  |  |  |  |  |  |
| S29 | 0.901 | 0.916 | 0.888 | 0.937 | 0.935 | 0.723 | 0.758 | 0.756 | 0.761 | 0.745 | 0.781 | 1 |  |  |  |  |  |  |
| S30 | 0.789 | 0.815 | 0.773 | 0.848 | 0.842 | 0.838 | 0.863 | 0.862 | 0.860 | 0.853 | 0.878 | 0.976 | 1 |  |  |  |  |  |
| S31 | 0.880 | 0.901 | 0.866 | 0.922 | 0.92 | 0.752 | 0.786 | 0.785 | 0.793 | 0.774 | 0.805 | 0.998 | 0.984 | 1 |  |  |  |  |
| S32 | 0.826 | 0.844 | 0.812 | 0.878 | 0.872 | 0.822 | 0.848 | 0.847 | 0.839 | 0.838 | 0.868 | 0.985 | 0.997 | 0.990 | 1 |  |  |  |
| S33 | 0.800 | 0.822 | 0.786 | 0.86 | 0.852 | 0.860 | 0.883 | 0.882 | 0.874 | 0.875 | 0.901 | 0.973 | 0.996 | 0.981 | 0.997 | 1 |  |  |
| S34 | 0.817 | 0.838 | 0.804 | 0.874 | 0.866 | 0.848 | 0.872 | 0.871 | 0.863 | 0.863 | 0.891 | 0.978 | 0.996 | 0.985 | 0.998 | 1 | 1 |  |
| S | 0.904 | 0.922 | 0.894 | 0.948 | 0.941 | 0.781 | 0.813 | 0.812 | 0.812 | 0.803 | 0.836 | 0.984 | 0.964 | 0.984 | 0.977 | 0.975 | 0.980 | 1 |

C

|  | S35 | S36 | S37 | S38 | S39 | S40 | S41 | S42 | S43 | S44 | S45 | S46 | S47 | S48 | S49 | S50 | S51 | S |
| --- | --- | --- | --- | --- | --- | --- | --- | --- | --- | --- | --- | --- | --- | --- | --- | --- | --- | --- |
| S35 | 1 |  |  |  |  |  |  |  |  |  |  |  |  |  |  |  |  |  |
| S36 | 1 | 1 |  |  |  |  |  |  |  |  |  |  |  |  |  |  |  |  |
| S37 | 0.993 | 0.994 | 1 |  |  |  |  |  |  |  |  |  |  |  |  |  |  |  |
| S38 | 0.993 | 0.994 | 1 | 1 |  |  |  |  |  |  |  |  |  |  |  |  |  |  |
| S39 | 0.995 | 0.995 | 0.995 | 0.995 | 1 |  |  |  |  |  |  |  |  |  |  |  |  |  |
| S40 | 0.943 | 0.945 | 0.972 | 0.972 | 0.965 | 1 |  |  |  |  |  |  |  |  |  |  |  |  |
| S41 | 0.953 | 0.955 | 0.978 | 0.978 | 0.973 | 0.999 | 1 |  |  |  |  |  |  |  |  |  |  |  |
| S42 | 0.941 | 0.943 | 0.970 | 0.971 | 0.964 | 1 | 0.999 | 1 |  |  |  |  |  |  |  |  |  |  |
| S43 | 0.936 | 0.939 | 0.967 | 0.967 | 0.961 | 1 | 0.998 | 1 | 1 |  |  |  |  |  |  |  |  |  |
| S44 | 0.938 | 0.940 | 0.968 | 0.968 | 0.962 | 1 | 0.999 | 1 | 1 | 1 |  |  |  |  |  |  |  |  |
| S45 | 0.943 | 0.945 | 0.971 | 0.971 | 0.965 | 1 | 0.999 | 1 | 1 | 1 | 1 |  |  |  |  |  |  |  |
| S46 | 0.965 | 0.966 | 0.978 | 0.979 | 0.967 | 0.974 | 0.976 | 0.971 | 0.969 | 0.969 | 0.974 | 1 |  |  |  |  |  |  |
| S47 | 0.945 | 0.946 | 0.955 | 0.956 | 0.943 | 0.948 | 0.95 | 0.944 | 0.942 | 0.942 | 0.948 | 0.993 | 1 |  |  |  |  |  |
| S48 | 0.960 | 0.961 | 0.972 | 0.973 | 0.955 | 0.955 | 0.957 | 0.952 | 0.949 | 0.950 | 0.955 | 0.996 | 0.993 | 1 |  |  |  |  |
| S49 | 0.965 | 0.967 | 0.984 | 0.984 | 0.972 | 0.984 | 0.985 | 0.982 | 0.980 | 0.981 | 0.984 | 0.998 | 0.986 | 0.991 | 1 |  |  |  |
| S50 | 0.960 | 0.962 | 0.975 | 0.976 | 0.962 | 0.968 | 0.970 | 0.965 | 0.963 | 0.963 | 0.968 | 0.998 | 0.995 | 0.998 | 0.997 | 1 |  |  |
| S51 | 0.938 | 0.940 | 0.956 | 0.957 | 0.938 | 0.958 | 0.957 | 0.954 | 0.952 | 0.952 | 0.958 | 0.995 | 0.995 | 0.996 | 0.990 | 0.996 | 1 |  |
| S | 0.974 | 0.976 | 0.991 | 0.991 | 0.984 | 0.991 | 0.993 | 0.990 | 0.988 | 0.988 | 0.991 | 0.992 | 0.973 | 0.982 | 0.997 | 0.989 | 0.977 | 1 |

D

|  | S6 | S7 | S8 | S9 | S10 | S11 | S23 | S24 | S25 | S26 | S27 | S28 | S40 | S41 | S42 | S43 | S44 | S45 | S |
| --- | --- | --- | --- | --- | --- | --- | --- | --- | --- | --- | --- | --- | --- | --- | --- | --- | --- | --- | --- |
| S6 | 1 |  |  |  |  |  |  |  |  |  |  |  |  |  |  |  |  |  |  |
| S7 | 1 | 1 |  |  |  |  |  |  |  |  |  |  |  |  |  |  |  |  |  |
| S8 | 1 | 1 | 1 |  |  |  |  |  |  |  |  |  |  |  |  |  |  |  |  |
| S9 | 0.999 | 1 | 1 | 1 |  |  |  |  |  |  |  |  |  |  |  |  |  |  |  |
| S10 | 1 | 1 | 1 | 1 | 1 |  |  |  |  |  |  |  |  |  |  |  |  |  |  |
| S11 | 1 | 1 | 1 | 1 | 1 | 1 |  |  |  |  |  |  |  |  |  |  |  |  |  |
| S23 | 0.998 | 0.998 | 0.999 | 0.998 | 0.999 | 0.999 | 1 |  |  |  |  |  |  |  |  |  |  |  |  |
| S24 | 0.998 | 0.999 | 0.999 | 0.998 | 0.999 | 0.999 | 1 | 1 |  |  |  |  |  |  |  |  |  |  |  |
| S25 | 0.998 | 0.999 | 0.999 | 0.998 | 0.999 | 0.999 | 1 | 1 | 1 |  |  |  |  |  |  |  |  |  |  |
| S26 | 0.998 | 0.999 | 0.999 | 0.998 | 0.999 | 0.999 | 1 | 1 | 1 | 1 |  |  |  |  |  |  |  |  |  |
| S27 | 0.998 | 0.998 | 0.999 | 0.998 | 0.999 | 0.999 | 1 | 1 | 1 | 1 | 1 |  |  |  |  |  |  |  |  |
| S28 | 0.999 | 0.999 | 1 | 0.999 | 1 | 1 | 1 | 1 | 1 | 1 | 1 | 1 |  |  |  |  |  |  |  |
| S40 | 0.998 | 0.998 | 0.999 | 0.999 | 0.999 | 0.999 | 0.999 | 0.999 | 0.999 | 0.999 | 0.999 | 1 | 1 |  |  |  |  |  |  |
| S41 | 0.998 | 0.998 | 0.999 | 0.999 | 0.999 | 0.999 | 0.999 | 0.999 | 0.999 | 0.999 | 0.999 | 1 | 1 | 1 |  |  |  |  |  |
| S42 | 0.998 | 0.998 | 0.999 | 0.999 | 0.999 | 0.999 | 0.999 | 0.999 | 0.999 | 0.999 | 0.999 | 0.999 | 1 | 1 | 1 |  |  |  |  |
| S43 | 0.997 | 0.997 | 0.998 | 0.998 | 0.998 | 0.998 | 0.999 | 0.998 | 0.998 | 0.998 | 0.999 | 0.999 | 1 | 1 | 1 | 1 |  |  |  |
| S44 | 0.997 | 0.997 | 0.998 | 0.998 | 0.998 | 0.998 | 0.999 | 0.999 | 0.998 | 0.998 | 0.999 | 0.999 | 1 | 1 | 1 | 1 | 1 |  |  |
| S45 | 0.998 | 0.998 | 0.999 | 0.999 | 0.999 | 0.999 | 0.999 | 0.999 | 0.999 | 0.999 | 0.999 | 1 | 1 | 1 | 1 | 1 | 1 | 1 |  |
| S | 0.999 | 0.999 | 1 | 0.999 | 1 | 1 | 0.999 | 1 | 1 | 0.999 | 0.999 | 1 | 1 | 1 | 1 | 0.999 | 0.999 | 1 | 1 |

**Table S2.** Precision, repeatability, and stability for three primary active ingredients in Ginger samples by HPLC-UV (280 nm)

|  | Precision(n=6) | | Repeatability(n=6) | | Stability(n=6) | |
| --- | --- | --- | --- | --- | --- | --- |
|  | Content  (μg/g) | R.S.D.  (%) | Content  (μg/g) | R.S.D.  (%) | Content  (μg/g) | R.S.D.  (%) |
| 6-gingerol | 380.72 | 0.14 | 365.33 | 0.69 | 360.34 | 3.19 |
| 8-gingerol | 82.52 | 1.98 | 56.73 | 5.85 | 63.78 | 1.94 |
| 10-gingerol | 257.11 | 0.71 | 154.60 | 3.97 | 160.19 | 3.37 |

| Repeatability(peak area) | | | |
| --- | --- | --- | --- |
| Sample No | 6-gingerol | 8-gingerol | 10-gingerol |
| CF-1 | 1641248 | 162152 | 254510.6667 |
| CF-2 | 1657022 | 164696.5 | 246651 |
| CF-3 | 1642166.333 | 164098 | 254729 |
| CF-4 | 1638789 | 183061 | 259402.6667 |
| CF-5 | 1658938 | 183775.3333 | 266229.6667 |
| CF-6 | 1666572.333 | 164079 | 275896.3333 |

| Precision(peak area) | | | |
| --- | --- | --- | --- |
| Sample No | 6-gingerol | 8-gingerol | 10-gingerol |
| JM-1 | 1719612 | 245454 | 433697 |
| JM-2 | 1719893 | 256041 | 433230 |
| JM-3 | 1721121 | 245858 | 433779 |
| JM-4 | 1720249 | 245728 | 433130 |
| JM-5 | 1721319 | 248050 | 432250 |
| JM-6 | 1726410 | 255605 | 440688 |

| Stability(peak area) | | | |
| --- | --- | --- | --- |
| Time(h) | 6-gingerol | 8-gingerol | 10-gingerol |
| 1 | 1606712 | 193021 | 415291 |
| 2 | 1611928 | 195019 | 412687 |
| 4 | 1603848 | 194004 | 424737 |
| 6 | 1603696 | 193807 | 423785 |
| 8 | 1580965 | 194861 | 428242 |
| 10 | 1601274 | 195241 | 413583 |
| 12 | 1601079 | 197268 | 425677 |
| 14 | 1672111 | 191621 | 400098 |
| 16 | 1663081 | 190843 | 428759 |
| 18 | 1632967 | 191350 | 457177 |
| 20 | 1596095 | 187159 | 429994 |
| 22 | 1608573 | 184600 | 412587 |
| 24 | 1780688 | 186674 | 439789 |

**Table S3.** Contents (μg/g) of three major active ingredients in different samples of ginger (n=3).

| Sample | Contents (μg/g) | | |
| --- | --- | --- | --- |
|  | 6-gingerol | 8-gingerol | 10-gingerol |
| S1 | 363.25 | 54.07 | 96.02 |
| S2 | 366.69 | 54.90 | 93.04 |
| S3 | 363.45 | 54.71 | 96.10 |
| S4 | 362.71 | 60.89 | 97.87 |
| S5 | 367.10 | 61.12 | 100.46 |
| S6 | 368.77 | 54.70 | 104.11 |
| S7 | 315.66 | 84.27 | 141.04 |
| S8 | 263.29 | 80.94 | 169.94 |
| S9 | 203.06 | 65.91 | 153.48 |
| S10 | 233.20 | 72.75 | 174.43 |
| S11 | 236.49 | 68.35 | 172.48 |
| S12 | 159.98 | 50.83 | 113.29 |
| S13 | 359.67 | 64.68 | 126.75 |
| S14 | 378.71 | 84.27 | 104.06 |
| S15 | 394.83 | 70.47 | 111.09 |
| S16 | 318.67 | 62.53 | 96.72 |
| S17 | 351.87 | 57.08 | 82.63 |
| S18 | 183.89 | 44.69 | 90.10 |
| S19 | 286.80 | 60.07 | 117.43 |
| S20 | 207.28 | 40.70 | 81.76 |
| S21 | 163.75 | 32.70 | 86.58 |
| S22 | 201.81 | 47.28 | 104.58 |
| S23 | 162.69 | 57.61 | 151.63 |
| S24 | 179.61 | 64.57 | 153.69 |
| S25 | 171.75 | 60.24 | 153.12 |
| S26 | 236.44 | 82.81 | 151.98 |
| S27 | 221.31 | 67.04 | 151.74 |
| S28 | 215.24 | 70.27 | 148.37 |
| S29 | 211.91 | 29.29 | 45.17 |
| S30 | 225.66 | 31.12 | 49.50 |
| S31 | 224.71 | 31.63 | 48.60 |
| S32 | 290.08 | 40.70 | 63.15 |
| S33 | 215.68 | 29.69 | 46.50 |
| S34 | 270.77 | 37.35 | 57.26 |
| S35 | 277.54 | 64.33 | 158.08 |
| S36 | 393.64 | 68.87 | 173.90 |
| S37 | 395.49 | 61.08 | 125.46 |
| S38 | 366.01 | 68.81 | 128.49 |
| S39 | 284.18 | 52.10 | 110.54 |
| S40 | 300.32 | 59.75 | 154.92 |
| S41 | 262.57 | 51.60 | 141.58 |
| S42 | 228.86 | 61.25 | 146.82 |
| S43 | 329.16 | 67.41 | 145.10 |
| S44 | 263.54 | 67.32 | 135.64 |
| S45 | 323.12 | 68.61 | 183.04 |
| S46 | 451.49 | 57.02 | 92.33 |
| S47 | 453.78 | 52.98 | 95.31 |
| S48 | 465.72 | 58.88 | 102.19 |
| S49 | 448.67 | 53.89 | 86.69 |
| S50 | 436.76 | 57.04 | 94.60 |
| S51 | 399.98 | 51.98 | 84.53 |

**Table S4.** Effect evaluation of discriminant function equation of superfine pretreatment.

| Statistical magnitude | Origin | Discriminant function prediction classification | | | Total |
| --- | --- | --- | --- | --- | --- |
|  |  | Shandong | Anhui | Yunnan |  |
| Number of samples | Shandong | 11 | 4 | 2 | 17 |
|  | Anhui | 2 | 14 | 1 | 17 |
|  | Yunnan | 4 | 0 | 13 | 17 |
| Percentage (%) | Shandong | 64.7 | 23.5 | 11.8 | 100.0 |
|  | Anhui | 11.8 | 82.4 | 5.9 | 100.0 |
|  | Yunnan | 23.5 | 0.0 | 76.5 | 100.0 |

**Table S5.** Effect evaluation of discriminant function equation of grinding pretreatment.

| Statistical magnitude | Origin | Discriminant function prediction classification | | | Total |
| --- | --- | --- | --- | --- | --- |
|  |  | Shandong | Anhui | Yunnan |  |
| Number of samples | Shandong | 16 | 1 | 0 | 17 |
|  | Anhui | 2 | 15 | 0 | 17 |
|  | Yunnan | 0 | 0 | 17 | 17 |
| Percentage (%) | Shandong | 94.1 | 5.9 | 0.0 | 100.0 |
|  | Anhui | 11.8 | 88.2 | 0.0 | 100.0 |
|  | Yunnan | 0.0 | 0.0 | 100.0 | 100.0 |

**Table S6.** Effect evaluation of discriminant function equation of liquid nitrogen pretreatment.

| Statistical magnitude | Origin | Discriminant function prediction classification | | | Total |
| --- | --- | --- | --- | --- | --- |
|  |  | Shandong | Anhui | Yunnan |  |
| Number of samples | Shandong | 12 | 4 | 1 | 17 |
|  | Anhui | 1 | 15 | 1 | 17 |
|  | Yunnan | 4 | 0 | 13 | 17 |
| Percentage (%) | Shandong | 70.6 | 23.5 | 5.9 | 100.0 |
|  | Anhui | 5.9 | 88.2 | 5.9 | 100.0 |
|  | Yunnan | 23.5 | 0.0 | 76.5 | 100.0 |

**Table S7.** Effect evaluation of discriminant function equation of Anhui.

| Statistical magnitude | Origin | Discriminant function prediction classification | | | Total |
| --- | --- | --- | --- | --- | --- |
|  |  | Superfine | Grinding | Liquid nitrogen |  |
| Number of samples | Superfine | 6 | 0 | 0 | 6 |
|  | Grinding | 0 | 6 | 0 | 6 |
|  | Liquid nitrogen | 0 | 0 | 6 | 6 |
| Percentage (%) | Superfine | 100.0 | 0.0 | 0.0 | 100.0 |
|  | Grinding | 0.0 | 100.0 | 0.0 | 100.0 |
|  | Liquid nitrogen | 0.0 | 0.0 | 100.0 | 100.0 |
